# High-purity isolation of rare single cells from blood using a tiered microchip system

**DOI:** 10.1371/journal.pone.0229949

**Published:** 2020-03-17

**Authors:** Onur Gur, Chun-Li Chang, Rohil Jain, Yuan Zhong, Cagri A. Savran

**Affiliations:** 1 School of Electrical Engineering, Purdue University, West Lafayette, IN, United States of America; 2 Birck Nanotechnology Center, Purdue University, West Lafayette, IN, United States of America; 3 School of Mechanical Engineering, Purdue University, West Lafayette, IN, United States of America; University of California San Diego, UNITED STATES

## Abstract

We present a two-tiered microchip system to capture and retrieve rare cells from blood samples with high purity. The first module of the system is a high throughput microfluidic interface that is used to immunomagnetically isolate targeted rare cells from whole blood, and discard > 99.999% of the unwanted leukocytes. The second module is a microwell array that furthers the purification by magnetically guiding each cell into a separate well concurrently, and allows individual retrieval of each cell. We demonstrate the design of the system as well as its characterization by experiments using model cell lines that represent circulating fetal trophoblasts. Our results show that single cells can be retrieved with efficiencies and purities as high as 100% within 145 mins.

## Introduction

Chromosomal abnormalities, including aneuploidy, translocations, dislocations and deletions occur in 1 in every 150 live births [1]. Current methods to diagnose these abnormalities include amniocentesis and chorionic villus sampling (CVS). These invasive procedures come with a risk of miscarriage; around 1% for amniocentesis and 2% for CVS [2–6]. To alleviate these difficulties, non-invasive prenatal diagnostics methods are being developed.

One commercially available method involves retrieval of cell free fetal DNA (cffDNA) from the blood plasma of the mother and analyzing it to detect genetic anomalies. While this method is effective in detecting a few conditions that include Trisomies 13, 18, and 21; the fragmented nature of the fetal DNA, and the contamination from maternal DNA makes it difficult to diagnose many other genetic disorders stemming from conditions such as mosaicism, small deletions, duplications or expansions [1,7].

An alternate non-invasive diagnostics method involves circulating fetal cells (CFCs). CFCs can be found as early as 6–8 weeks into pregnancy and can be retrieved from maternal blood without risking the fetus or the mother [8]. They are more effective in diagnosing chromosomal abnormalities in fetuses compared to cffDNA due to their intact fetal genome, and lack of contamination from maternal DNA [9]. The major challenge regarding CFCs is that they are extremely rare, ranging from 1–2 cells per milliliter of blood [10,11]. This has led to the development of several isolation methods for CFCs over the years. We provide below a detailed summary of existing methods, recent developments as well as the ensuing opportunities for improvement.

Conventional methods for CFC enrichment include fluorescence activated cell sorting (FACS), magnetic activated cell sorting (MACS), and methods based on the size of the cell such as density gradient centrifugation and filtration [2,12].

FACS and MACS are methods that rely on specific biomarkers that target cells express to separate them from a sample fluid. They both result in relatively low purity, i.e. a great number of unwanted cells which could necessitate additional enrichment steps. For example, a study by Bianchi et al., where 20 ml of maternal blood was enriched for cells that express the transferrin receptor (TfR), yielded between 46,000 to 673,000 TfR^+^ cells; of which an average of only 150 were determined to be the targeted cells by subsequent PCR and Southern blot analyses [13]. Experiments performed by Chen et al. where 20 target cells were spiked into 5 ml blood showed that negative enrichment by MACS result in recovery rates of around 35% with a total number of 27900 cells [9]. Hatt et al. used MACS by targeting the marker set CD105 and CD141 which resulted in 500,000 cells, only 0 to 18 of which were classified as candidate fetal cells after fluorescent labeling and manual scanning of the cells [14].

Density gradient separation, where cells are suspended in a solution with density gradient also have purity levels that are generally low. Two studies by Calabrese et al. in 2011 and 2016 on fetuses with aneuploidy yielded a total of 50,000–100,000 cells of which only 4–9 were target cells per 25 ml blood, and 160,000–220,000 cells of which only 4–34 were target cells per 24 ml blood respectively [15,16].

Multiple groups used size-based detection to target CFCs. Vona et al. used polycarbonate filters with 8 μm to target CFCs and Mohamed et al. used successively narrowing channels to separate CFCs based on their size and deformation characteristics [17,18]. These filtration methods rely on the assumption that there are significant size and deformity differences between the targeted cells and other cells, which is not necessarily the case at all times.

Recently, various microfluidic devices were developed to further advance CFC isolation. Byeon et al. used a 2-step enrichment process to increase the purity of retrieved CFCs. A red blood cell hyperagregation step was used to facilitate the removal of white blood cells (WBCs) remaining in the supernatant ; followed by further purification by negative enrichment using a lateral magnetophoretic microseparator [19]. He et al. used an affinity based nanostructure microchip to isolate and perform in situ analysis of CFCs [20] whereas Hou et al. developed the nano-Velcro microchip to isolate CFCs followed by laser capture microdissection (LCM) to retrieve them [21]. Huang et al. used a Si based microchip utilizing immunoaffinity to capture and retrieve CFCs [22]. Commercially, RareCyte developed a system which was demonstrated by Breman et al. where CFCs were isolated from blood, imaged automatically, and retrieved using a semi-automated picking routine [23]. A common limitation of most microfluidic systems is that they generally cannot directly process a standard tube of blood sample (~8 ml) without an initial volume reduction step. This volume reduction is achieved either by centrifugation, which can lead to losses in cell viability, function, and number [2,12]; or red blood cell hyper aggregation, which can lead to losing precious target cells [19].

An additional common limitation in affinity based cell capture methods is the usage of a single antibody [20–22]. This hinders a platform’s ability to capture cells that may not have a reduced expression of the targeted single antigen. Targeting a cocktail of antigens within the same device would mitigate this problem and circumvent the need to fabricate a different device for each antigen.

Upon successful initial isolation of target cells from a complex fluid, a critically important next step is to retrieve the cells individually for downstream analysis [19,20]. Most existing platforms release all captured cells from their surface in bulk into a single container. This hinders most types of analyses because the captured group of cells often contains non-targeted cells such as WBCs which reduces the overall purity by posing as background. Retrieving individual cells is therefore challenging since avoiding WBCs that surround a target cell often requires multiple attempts [23].

In this paper, we present a system that primarily aims to overcome the aforementioned shortcomings related to purity. In addition, the system minimizes the sample preparation steps by processing a whole blood sample directly, without the need of any centrifugation, and is versatile enough to target a variety of antigens to achieve high recovery rate of cells. Even though the system we have developed is applicable to many fields pertaining to rare cell capture, here we demonstrate the proof-of-concept using model cell lines that represent circulating fetal trophoblasts. Extravillous trophoblasts represent the genotype of the fetus (except in rare cases (1%) of confined placental mosaicism) and are expected to make an impact on the future of non-invasive prenatal diagnostics [2,21,23]. We describe the design, operation as well as the experimental characterization of the system. Our results show that the process can be completed within 145 minutes from the very beginning till the retrieval of a target cell, and can provide efficiencies and purities that are as high as 100%.

### Isolation system and single cell retrieval strategy

**Fig 1:**
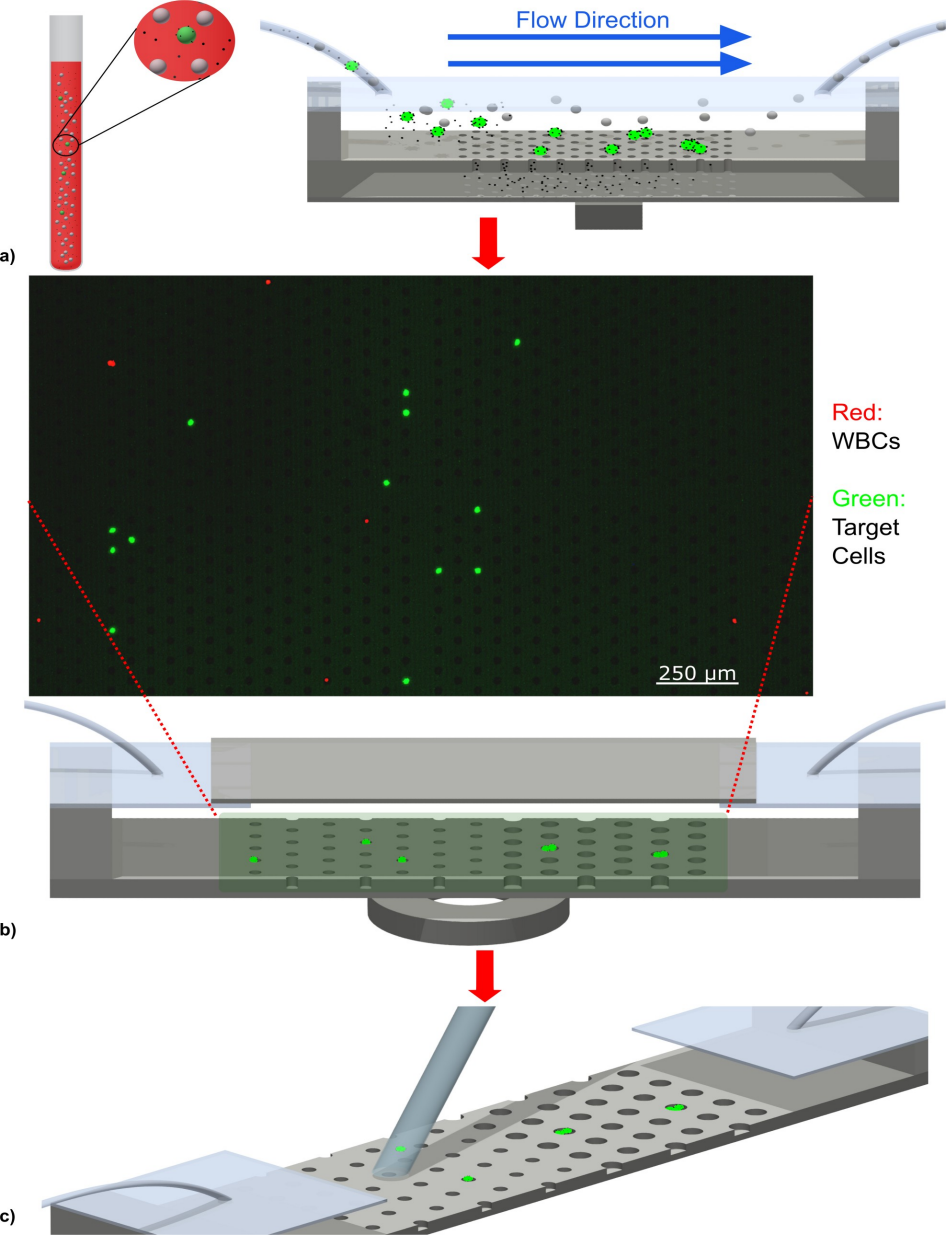
a) Isolation of target cells on porous chip. b) The target cells after they are transferred to well chip and separated in different wells. c) Retrieval of target cells using a micro pipette.

Our strategy involves two distinct microchip modules used in three steps. The first step is the isolation of the target cells from blood using a “porous chip” which we introduced previously [24]. Briefly, magnetic beads conjugated with antibodies are incubated with a blood sample to bind the surface antigens of the target cells. The mixture is then flowed through a fluidic chamber that contains a porous microchip that magnetically captures the target cells, clears out excess magnetic beads and washes away bulk of the unwanted white blood cells (WBCs) (Fig 1A).

The first step discards nearly 99.999875% of the WBCs. However, due to non-specific binding of the magnetic beads to WBCs, there are always some 100s of WBCs captured by the porous chip alongside the targeted rare cells. Any target cells retrieved at this stage would be mixed with WBCs, reducing the purity. An attempt to aspirate the targeted cells at this stage would also be problematic since all cells on the chip surface, including the unwanted WBCs would be retrieved which would also reduce the overall purity.

Hence, in order to increase the purity further, the cells captured on the porous chip are transferred to a second module which accommodates a “well chip” which compartmentalizes all cells and allows their individual retrieval. The release of the cells from the porous chip is achieved by removing the magnet from underneath the porous chip, and initiating a buffer flow. Cells are next captured on the well chip surface once again by providing a magnetic field from below. Sweeping this magnetic field horizontally downstream directs the cells towards an array of microwells. Once a cell encounters a microwell, it falls into the microwell due to the downward magnetic force (Fig 1B).

The diameter of microwells increase as cells move downstream. Hence, if a cell (or a group of cells) is too large to fit inside a microwell, it will eventually encounter and fall into a larger well downstream. This scheme not only enables a simple size-based classification of captured cells but also allows isolating each cell from all other cells, including those that were captured on the porous chip nonspecifically. Once each cell is captured in an individual well, the well chip is imaged via immunofluorescence to analyze and identify the location of the targeted cells (Fig 1B).

Finally, the top cover of the fluidic chamber that accommodates the well chip is opened up to retrieve each targeted cell with a micro pipette (Fig 1C).

In order to characterize the operation of the system, we used JEG3 cells, a human choriocarcinoma cell line. The JEG3 has a surface antigen expression that is similar to that of circulating fetal trophoblasts and is frequently used in similar system characterization/optimization experiments [21,25].

To recognize JEG3 cells, we functionalized magnetic beads with antibodies against EpCAM (Epithelial Cell Adhesion Molecule) as well as HLA-G (Human Leukocyte Antigen G); surface antigens that are commonly used to target/verify circulating fetal trophoblasts [17,21,22,26–28]. These two antigens are commonly used due to 1) the epithelial nature of trophoblasts which warrants the targeting of EpCAM; and 2) the role of HLA-G in preventing the rejection of the trophoblasts by the maternal immune system [17,29]. The lack of these antigens on WBCs prevent the specific attachment of the functionalized beads on them. Functionalized beads were incubated with blood samples to bind to targeted cells, and the mixture was then run through the fluidic chamber that contains the porous chip with a pore dimeter of 6 μm. The well chip contained 4 groups of wells whose diameters increased from upstream to downstream: 15, 20, 30 and 60 μm. Such an arrangement ensured capturing single cells as well as clusters of cells. The wells had a uniform well depth of 20 μm which was sufficient to retain the cells in the wells and prevent them from escaping under the influence of flow. The depth of the wells was deliberately limited so as to minimize the probability of losing a cell in a well that is too deep, and to facilitate extraction by micro-pipetting. More detail on the well chip design can be found in the results section.

The immunofluorescence confirmation of the cells was accomplished by using anti-EpCAM-FITC, and anti-HLA-G-FITC antibodies. This was performed to verify that the captured cells are indeed EpCAM and/or HLA-G+. Hoechst staining was used to verify that the cells had nuclear DNA, and anti-CD-45-PE antibodies were used to identify and rule out WBCs. The choice of Hoechst allowed keeping the cells alive (vs. DAPI which requires that the cells are fixed and permeabilized). We used a micro pipette with a 50 μm tip to retrieve the cells from the well chip.

## Materials and methods

### Fabrication of the chips

8 porous chips were fabricated on a 4 inch diameter, 500 μm thick <100> oriented double side polished Silicon wafer using the process described in a previous publication [30].

**Fig 2:**
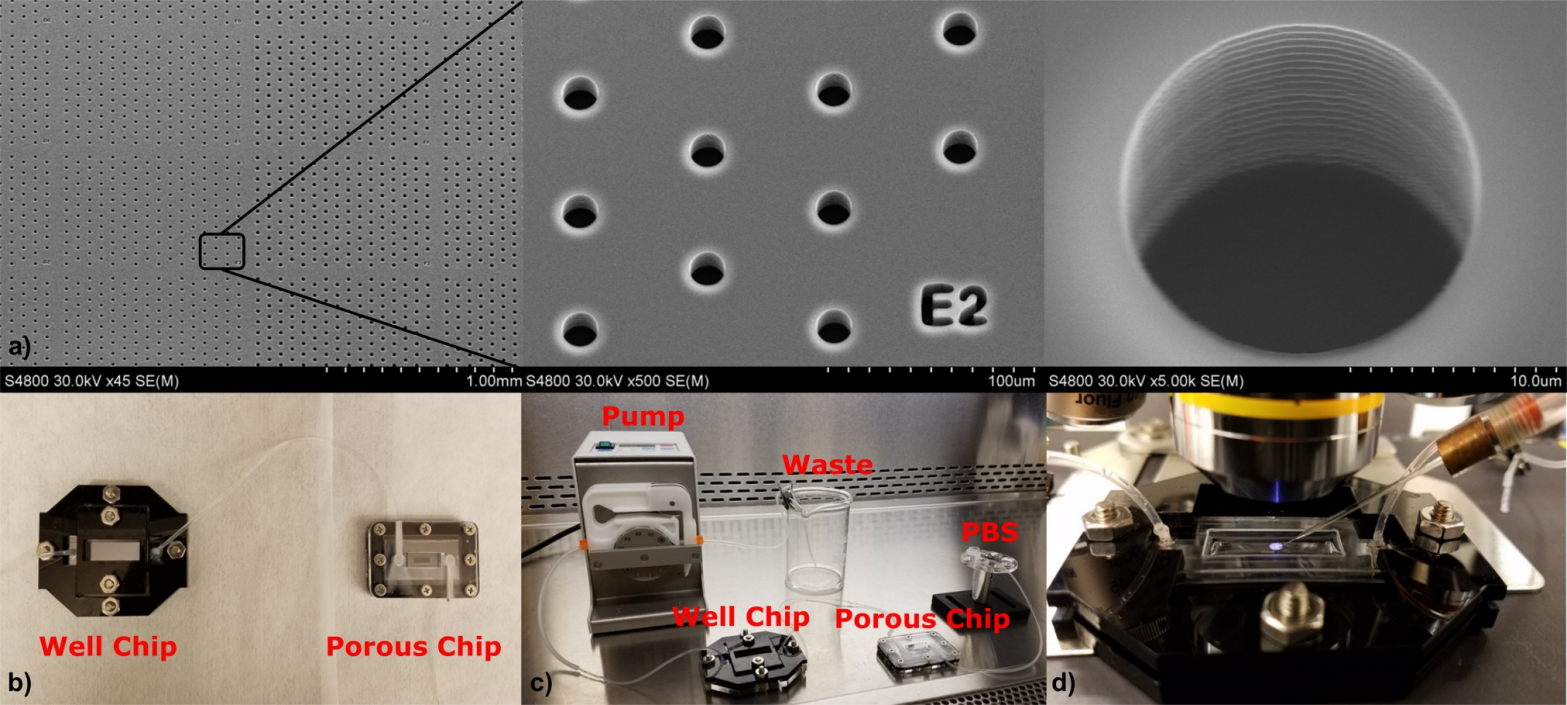
a) SEM images of the wells. b) Image of porous chip and well chip inside microfluidic chambers. c) The bench setup while transferring cells from porous chip to well chip. d) The setup used for picking cells with the micro pipette.

For the well chip, a 500 μm thick <100> oriented single side polished Si wafer was coated with positive photoresist (Microchemicals, AZ9260) to define the areas with wells. To etch the wells, Deep Reactive Ion Etching (Surface Technology Systems, ASE) was used to obtain the 20 μm deep wells. The remaining photoresist was removed with a photoresist stripper (Baker, PRS-2000). This process resulted in 4 well chips in a wafer, each with the dimensions of 51mm*26mm. The inner area that contained the micro wells measured 20mm*7mm as seen in Fig 2A.

After the fabrication, both chips were coated with a zwitterionic sulfobetaine methacrylate (SBMA) polymer, by using atom transfer radical polymerization (ATRP) to minimize the non-specific binding of impurities, using a method described by Brault et al [31].

### Experimental setup

The porous chip and the well chip were housed in 2 different microfluidic chambers (Fig 2B). For the porous chip, the fluidic chamber was defined by a 1 mm thick PDMS with a 3.8 mm by 30 mm groove. The top was covered by a 1 mm thick glass and the bottom was sealed by a 0.1mm thick PDMS and a transparency sheet (3M PP2500, 0.1 mm thick). The structure was sandwiched between a top and bottom 3mm thick acrylic sheet (Grainger, 1UNZ5) to ensure the sealing of the entire chamber.

For the well chip, a 1mm thick PDMS with a grove of 10mm by 46 mm was used to define the fluidic chamber. On top of this, a 0.5 mm thick PETG (Polyethylene Terephthlate Glycol-Modified) Sheet (Small Parts, VIS-060335-G-01) with a grove of 10mm by 30mm was used as a top cover. The groove on the PETG was sealed by a 2mm PDMS, which can be peeled off to access the chamber. This structure was sandwiched between a 2-piece 3 mm thick top acrylic and a 4.4 mm thick bottom acrylic (Grainger, 1UNZ5 and 1UNZ6). The top acrylic had an outer piece that sealed the fluidic chamber while the inner part was made removable to access the cells for retrieval.

A laser cutter (Universal Laser System, Inc. Professional Series) was used to cut all the grooves on acrylic and PETG. A drill was used to define the inlet and outlet of the glass top for the porous chip chamber. Both chambers were washed with DI water, RBS detergent (Thermo Fisher Scientific, 27950), ethanol (Thermo Fisher Scientific, A405P-4) and PBS (Thermo Fisher Scientific, 10010023) before being exposed to biological samples.

To capture cells from blood, a peristaltic pump (Ismatec, ISM596B) was used to move the sample fluid through the fluidic chamber that accommodated the porous chip. A N52 grade magnet (K&J Magnets, B444-N52) was placed to pull the target cells and the rest was collected at a waste container.

While transferring the cells from the porous chip to well chip, the outlet of our porous chip was connected directly to the inlet of the well chip, and the magnet under the porous chip was removed. A N52 grade ring magnet (with a maximum residual flux density Br_max_ of 14,800 Gauss) was placed under the well chip (K&J Magnets, R842-N52) and the outlet of the well chip was connected to the pump (Fig 2C).

A fluorescent microscope (Nikon, ECLIPSE 80i) was used to identify the target cells while a micro pipette (Clunbury Scientific, B100-58-50) was used to retrieve the desired cells (Fig 2D).

### Culturing and spiking of the cells

JEG3s were purchased from American Type Culture Collection (ATCC, HTB-36), and cultured in Eagle’s Minimum Essential Medium (EMEM) (ATCC, 30–2003) with 10% Fetal Bovine Serum (FBS) (ATCC, 30–2020). The cells were harvested with Trypsin-EDTA (ATCC, 30–2101), and suspended in media on a petri dish. For spiking, the cells were placed on a petri dish under a microscope and picked and spiked into blood using a micro pipette.

The blood was purchased from Biochemed Services (10761WB-EK2-FI), which had been retrieved from a single donor in a 100 ml container with EDTA anti-coagulant. The blood was stored at 4°C until cell spiking. ~8 mL aliquots of this blood sample were used in all experiments. EDTA-containing blood collection tubes may be used if the blood sample that contains live target cells will be processed relatively quickly (hours). However, if the blood sample containing target cells need to be stored for an extended period of time (days), alternative blood collection tubes that contain preservatives (e.g. those provided by CellSave, Streck, Norgen) can be used to minimize the uncertainties that could result from possible degradation of target cells.

### Detecting JEG3 cells spiked into blood

For each blood sample 2 antibody-bead complexes were prepared. 17.5 μl of biotinylated anti-EpCAM antibodies (R&D Systems, BAF960) with a concentration of 0.2 mg/ml was incubated with 14 μl of streptavidin beads (Thermo Fisher Scientific, 65601) with a concentration of 10 mg/ml, and suspended in 140 μl of PBS. Also, 2 μl biotinylated anti-HLAG antibodies (Thermo Fisher Scientific, MA1-10361) with a concentration of 1 mg/mL was incubated with 8 μl of streptavidin beads (Thermo Fisher Scientific, 65601) with a concentration of 10 mg/ml, and suspended in 80 μl of PBS. Both incubations took 1 hour and were followed by a triple wash with PBS to remove any unconjugated antibodies.

The antibody-bead mixtures were incubated with 8 ml of blood for 35 mins. Afterwards the sample was diluted to 16 ml by adding 8 ml of PBS, and was run through the porous chip. The suspension was run though the porous chip 2 times, once using a flow rate of 2 ml/min and then again using 1 ml/min. The high volumetric throughput capability of the system facilitated the recirculation. This was then followed by a 4 ml PBS wash using a 1ml/min flow rate. After the wash, any remaining red blood cells were removed by a 10 minute incubation with a RBC Lysis buffer (Biosciences, 786–672) followed by a 2 min PBS wash.

### Immunofluorescence analysis

**Fig 3:**
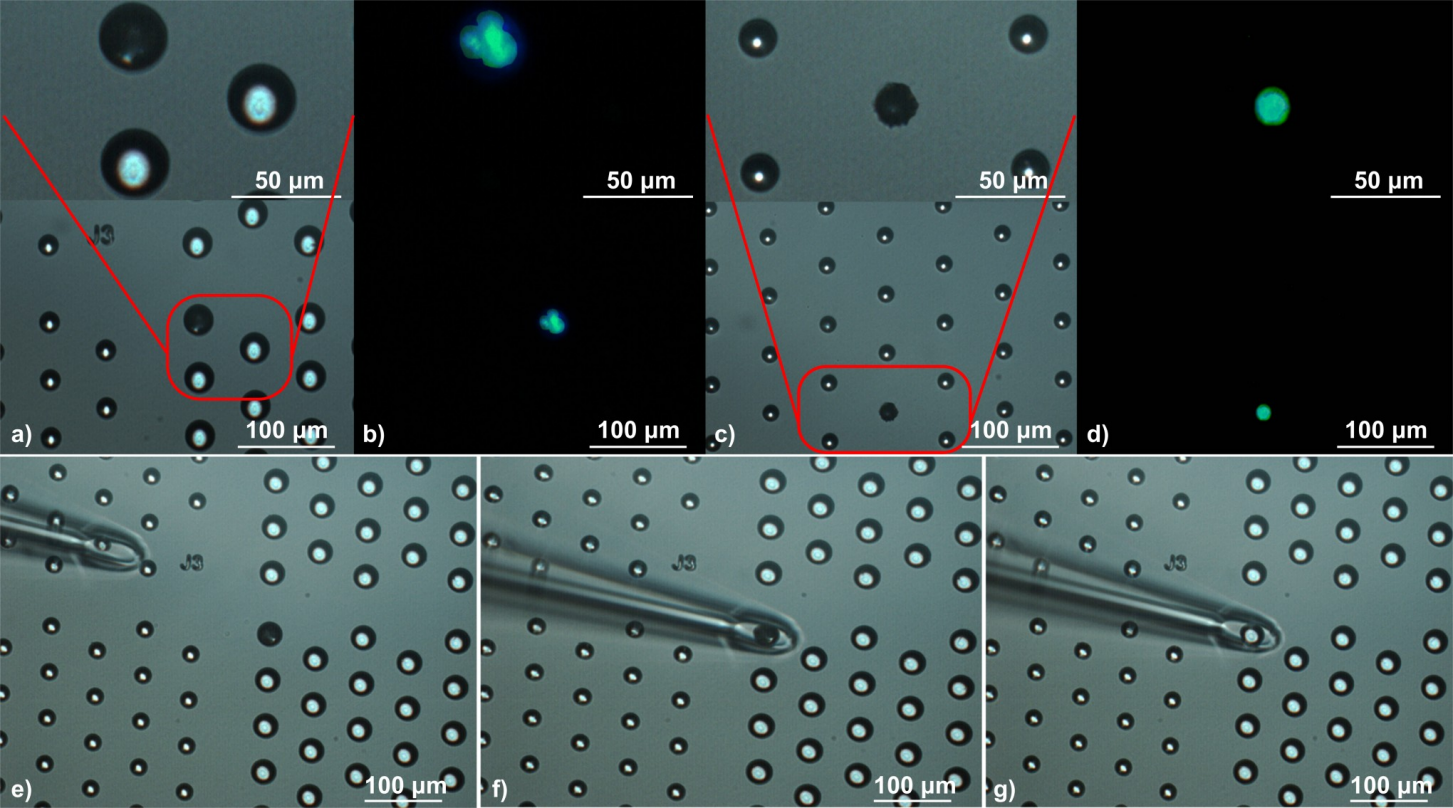
a) Bright field image of a 3 cell-cluster in a 30 μm diameter well. b) Combined anti-HLAG-FITC, anti-EPCAM-FITC, Hoechst image of the cells from Fig 3A. c) Bright Field image of a single cell in a 15 μm diameter well. d) Combined anti-HLAG-FITC, anti-EPCAM-FITC, Hoechst image of the cell from Fig 3C. e-g) Approaching and picking up of the cells from Fig 3A-3B with the micro pipette.

Before being transferred to the well chip for analysis and retrieval, the cells were stained while they were on the surface of the porous chip. The cells were initially stained with a mixture of anti-EpCAM-FITC (Miltenyi Biotech, 130-113-263), anti-HLAG-FITC (Miltenyi Biotech, 130-111-850), and anti-CD45-PE (Miltenyi Biotech, 130-113-118) antibodies for 15 min followed by a 4 ml PBS wash. Then, the cells were also stained with a Hoechst Solution (Thermo Fisher Scientific, 62249) for 10 mins followed by another 4 min PBS wash. This was followed by the transferring of the cells to the well chip. We identified JEG3 cells as EpCAM and/or HLAG + as well as Hoechst + (Fig 3A–3D). We identified WBCs as CD45 and Hoechst + . We adopted this protocol to retrieve live cells without fixation and permeabilization, a process often used to label cytokeratin [26,21,22,32].

### Transferring JEG3 cells to well chip

In order to transfer the cells to the well chip, the magnet underneath the porous chip was removed, and the outlet of the porous chip was connected to the inlet of the well chip. The contents of the porous chip were washed into the well chip via a 2 ml PBS wash at a flow rate of 2ml/min. The cells were captured on the well chip using a ring shape magnet underneath which helped spread the cells apart from each other. When the cells first contacted the well chip surface, they all were in the vicinity of the 15 μm wells. In order to move the cells that did not initially fall into a well, as well as to move larger cell clusters into to larger wells located downstream, a magnet was brought in close proximity (approx. 2 mm) of the bottom of the well chip and slowly moved downstream. This caused all cells to slide on the surface of the well chip until they found a well that they could fit into. Larger clusters that did not fit into the first group of wells (15 μm), continued sliding downstream until they reached 20, 30, and 60 μm wells (S1 Video).

### Retrieval of JEG3 cells from the well chip

After identifying the target cells on the well chip according to their fluorescent properties, their locations were recorded. Once the location of a cell is recorded, the fluorescence is no longer needed and the extraction can easily be performed in brightfield. Single cells were retrieved using a micro pipette with a 50 μm opening and manipulating it using a translation stage (Fig 3E–3G, S2 Video).

## Results and discussion

### Total time of assay

In addition to its overall simplicity and compatibility with standard laboratory equipment, an important strength of our system is its overall speed. A single cell can be isolated from an 8 ml tube of blood and extracted in 145 mins (Table 1).

**Table 1:** The timeline for the cell isolation and retrieval process.

Process	Hands On Time (min)	Total Time (min)
**Incubating the target cells with Ab-Bead complex.**	**1**	**35**
**Detecting cells on porous chip.**	**6**	**42**
Diluting blood with PBS.	2	2
Circulating the solution through the pump at 2ml/min.	1	8
Passing the solution through the pump at 1ml/min.	1	16
PBS wash.	1	4
RBC Lysis and wash.	1	12
**Immunofluorescence analysis.**	**4**	**33**
A-EPCAM-FITC, A-HLA-G-FITC, and A-CD45-PE staining and wash.	2	19
Hoechst staining and wash.	2	14
**Transferring and locating cells on well chip.**	**31**	**32**
Transfer from porous chip to well chip.	1	2
Locating cells on the well chip.	30	30
**Picking up a cell from well chip.**	**3**	**3**
**Total**	**45**	**145**

### Well chip system design and modeling

**Fig 4:**
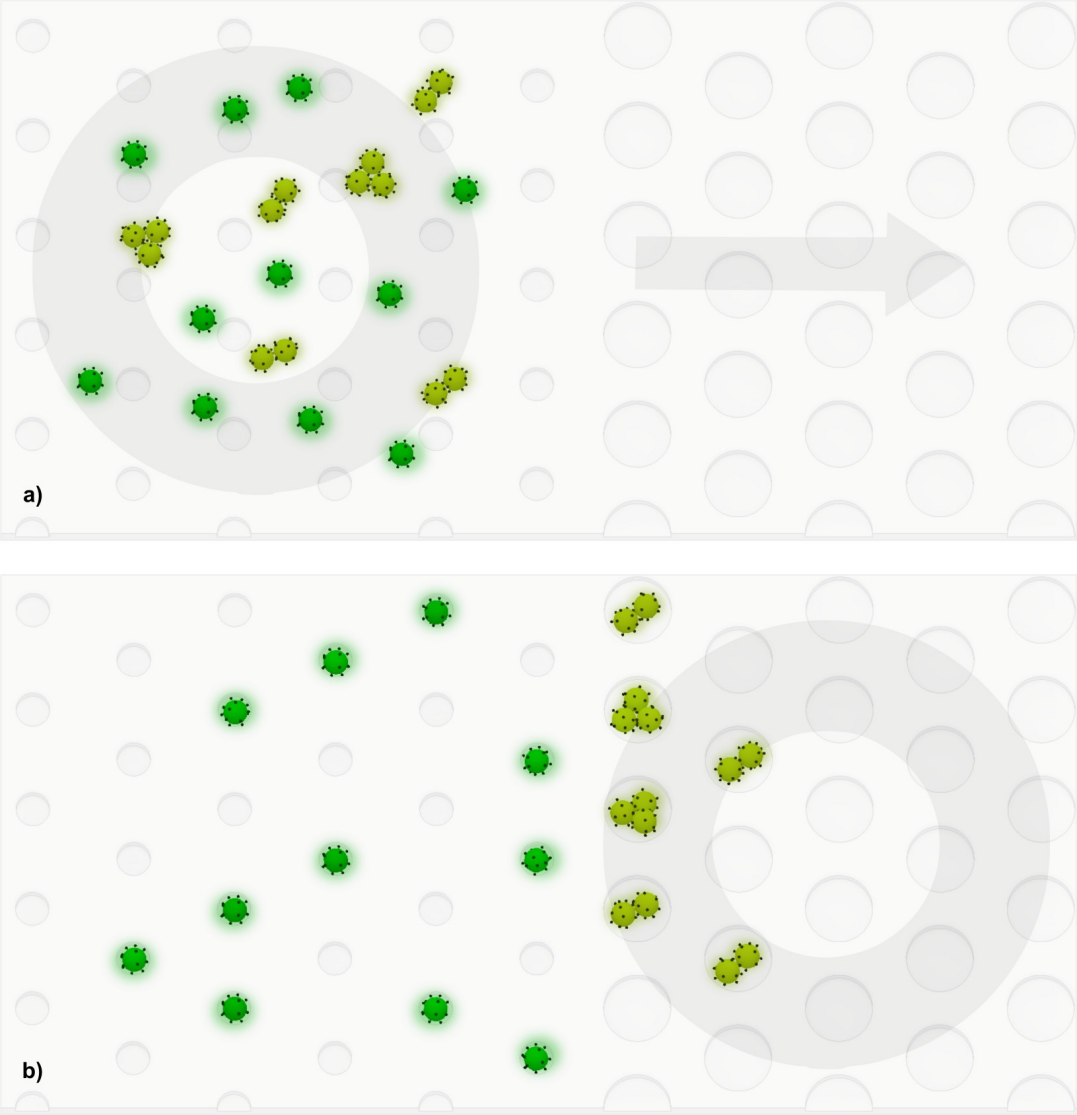
Movement of cells under the influence of the magnet. a) Before magnet sweep: The magnet is upstream (to the left) and the cells are distributed randomly. b) After magnet sweep: Magnet has moved downstream (to right) and cells and clusters are distributed into individual wells.

An important feature of the well chip operation is its robustness and overall simplicity. All cells and cell clusters are distributed into their respective wells simultaneously, and separated by size using only a horizontally moving magnetic field. This is achieved manually by the user moving the magnet by hand (Fig 4A and 4B). This leads to a quick and robust process that does not require monitoring cell motion under a microscope. We describe below various parameters that influenced its design and performance.

**Fig 5:**
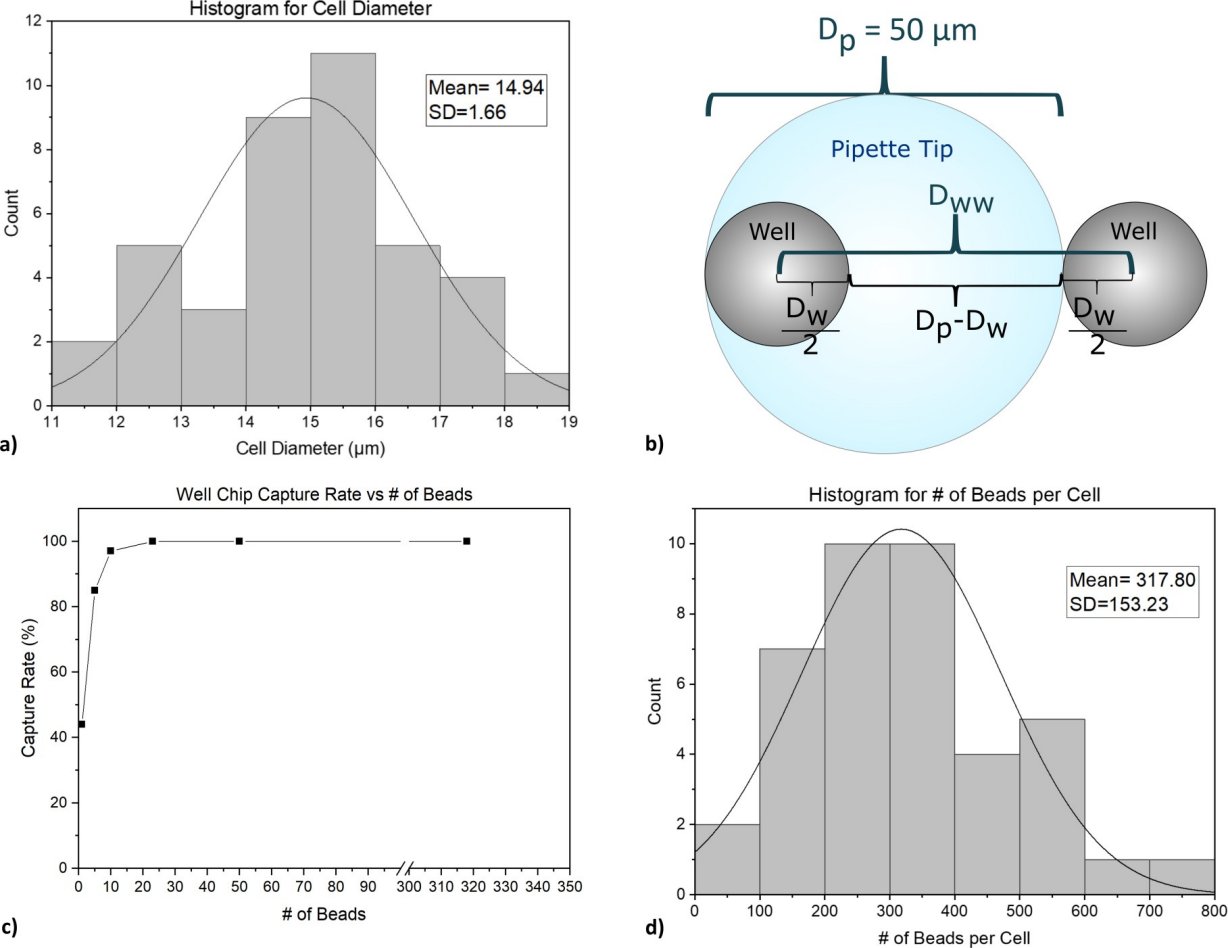
a) The distribution of the cell diameters. b) The distance between wells in relation to pipette tip. c) Detection rate on well chip vs the number of beads on a cell. d) The distribution of # of beads per cell.

**Well size.** In order to determine the size of the wells, the diameters of the 40 cultured JEG3 were recorded by microscopic observation. The diameters of JEG3s ranged from 11.3 μm to 18.3 μm (Fig 5A). The well sizes were accordingly chosen to be 15, 20, 30, and 60 μm. The 15 μm and 20 μm wells were designed to hold single cells while 30 μm and 60 μm wells were to hold cells in clusters. The depth of the wells was chosen as 20 μm which is deep enough to hold a single layer of cells inside.

### Well-to-well distance

The distance between neighboring wells, D_ww_ was determined based on the dimeter of the micro pipette tip that was used (Fig 5B) with the primary design goal being that the tip cross-section should only coincide with a single well while minimizing the distance between wells. Assuming a conservative scenario where the pipette tip fully covers a well while being tangent to a neighboring well (Fig 5B), and D_w_ (diameter of the well) to be less than D_p_ (the diameter of the pipette tip), we determined the minimum well-to-well distance to be equal to the diameter of the pipette tip which for our setup was 50 μm:

An additional 10 μm was added to account for uncertainties and 60 μm was chosen as the final value of D_ww_.

### Magnet type

In order to systematically determine the most appropriate type of magnet for distributing cells into individual wells, a few different commercial magnets were investigated via a numerical simulation. The particle tracing module of COMSOL was used to calculate the cell-to-cell separation of 100 target cells introduced into a channel with a width of 10 mm, and thickness of 1mm [24], under a fluidic flow rate of 2 ml/min.

The forces that primarily influence the target cells, namely magnetic and fluidic drag forces were calculated. Other forces such as gravity and buoyancy forces were ignored due to being at least 2 orders of magnitude lower.

The force on magnetic beads (Thermo Fisher Scientific, MA1-10361) was expressed using the following equation which is derived from the magnetic force acting on a point-like magnetic dipole [33]:

$$\vec{F_{m}}=N\frac{V_{p}\chi }{2\mu_{0}}\nabla (\vec{B}∙\vec{B})$$

Here *N* is the number of beads on a cell. A minimum of 23 beads per cell were needed to ensure 100% capture rate on well chip (Fig 5C, determined by COMSOL). By viewing the black spots on the cells under bright field microscopy, it was determined that the cells had an average of 318 beads per cell. This method of counting the beads was possible due to the relatively large size of the beads (1 μm) (Fig 5D). V_p_ is the volume of the bead (4.19 μm^3^), χ is the relative susceptibility (0.35) [34,35], B is the magnetic flux density [T] (calculated in COMSOL based on magnet properties), and μ_0_ is the vacuum permeability (1.257∙10^−6^ H/m).

The drag force on a cell was expressed by the following Stokes’ drag equation [36]:

$$\vec{F_{d}}=6\pi \mu R\upsilon$$

where μ is the dynamic viscosity (1.05∙10^−3^ Pa∙s for PBS), R is the radius of the cell, (7.5 μm), and *υ* is the flow velocity relative to the cell (calculated in COMSOL).

**Fig 6:**
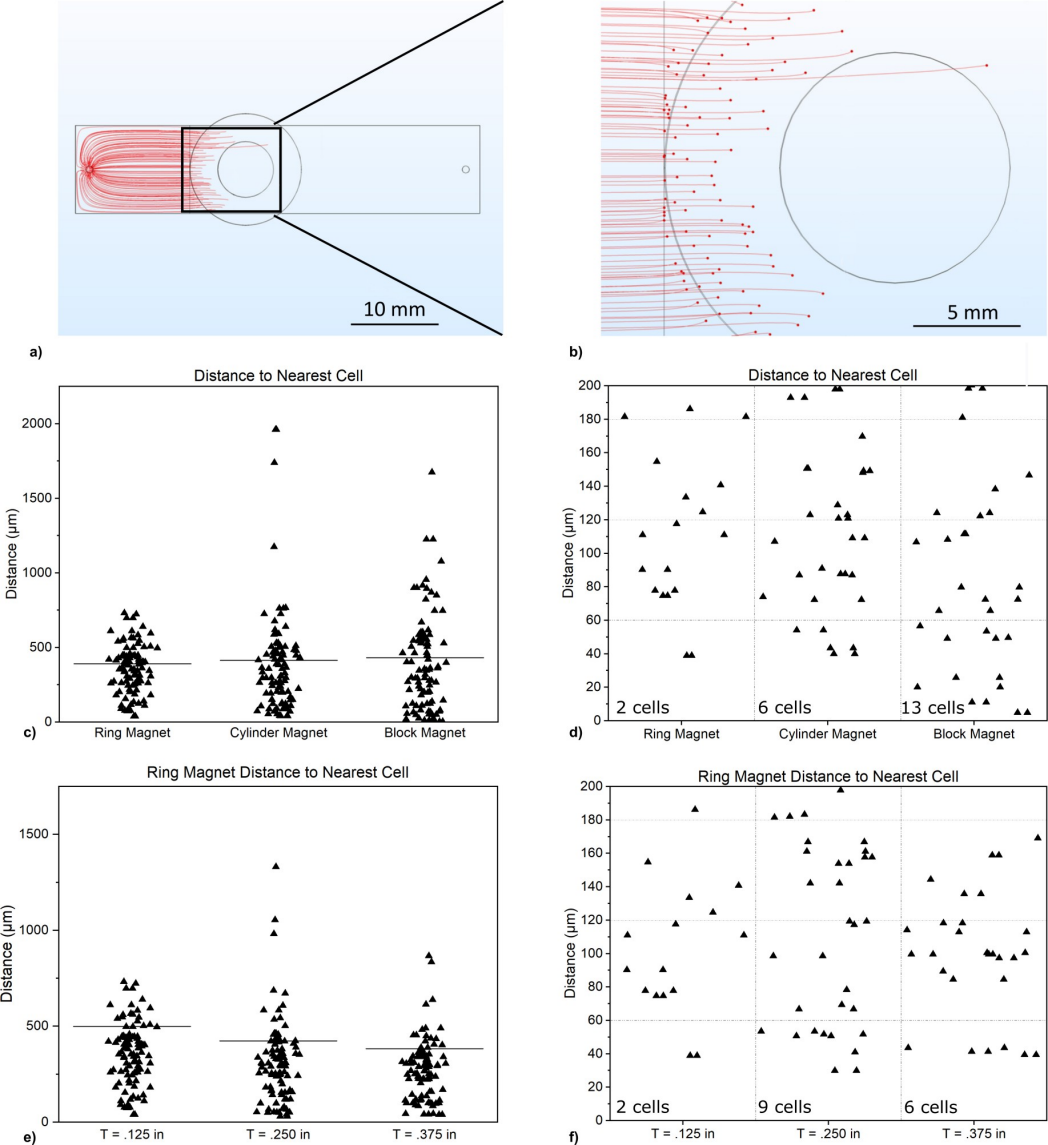
a) Distribution of the cells on the well chip with a ring magnet underneath. b) Zoomed in image of distribution of the cells on the well chip with a ring magnet underneath. c) Distance between nearest cells for ring, cylinder, and block magnets. Each data point indicates the distance between a cell and the cell nearest to it. d) Distance between nearest cells for ring, cylinder, and block magnet for distances <200 μm. e) Distance between nearest cells for ring magnets of varying thicknesses. Each data point indicates the distance between a cell and the cell nearest to it. f) Distance between nearest cells for ring magnets of varying thicknesses, for distances <200 μm. Horizontal lines on c and e indicate mean values.

The particle tracing module was used to trace the movement of the cells under the drag force and the magnetic force. Coordinates of the cells when they first touch the surface of the chip due to the pull of the magnetic force were recorded. The screenshot of a typical simulation, where the cells were tracked from the instant they were introduced from the inlet of the fluidic chamber to the instant they were pulled to the surface of the chip by a ring magnet is shown in Fig 6A and 6B.

Magnets that resulted in the greatest number of cells with larger than 60 μm cell-to-cell separation were preferred since that would indicate that the cells are more likely to encounter a well (since D_ww_ = 60 μm) when they reach the surface instead of encountering each other. A ring (0.5 in OD, 0.25 in ID, 0.125 in T), cylinder (0.5 in D, 0.125 in T), and a block (0.5 in L, 0.5 in, W, 0.125 in T) magnet were simulated. For each case, the distance between each cell, and the cell nearest to it are plotted. The simulation results revealed that 98%, 94%, and 87% of the cells would be more than 60 μm apart from the most adjacent cell for each magnet type respectively (Fig 6C and 6D). It was also observed by simulation that using thicker magnets would lead to more aggregation of cells as confirmed by the simulations with a ring magnet (0.5 in OD, 0.25 in ID) with varying thicknesses (0.125 in, 0.250 in, 0.375 in). The simulation results revealed that 98%, 91%, and 94% of the cells would be more than 60 μm apart from the most adjacent cell respectively (Fig 6E and 6F). Therefore, a ring magnet (K&J Magnets, R842-N52, 0.5 in OD, 0.25 in ID, 0.125 in T) was used for the experiments.

### System characterization

In order to characterize the performance of our system, we performed spiking experiments with 8 ml blood samples. We performed experiments using i) 10 single cells, ii) 25 single cells, and ii) 25 cells that contained known numbers of single cells as well as clusters of pairs, triplets and quadruplets. Each set of experiments was performed 3 times. We compared the number of cells we spiked into a blood sample to that we retrieved from the well chip after the blood sample went through all of the processes described in Table 1.

**Fig 7:**
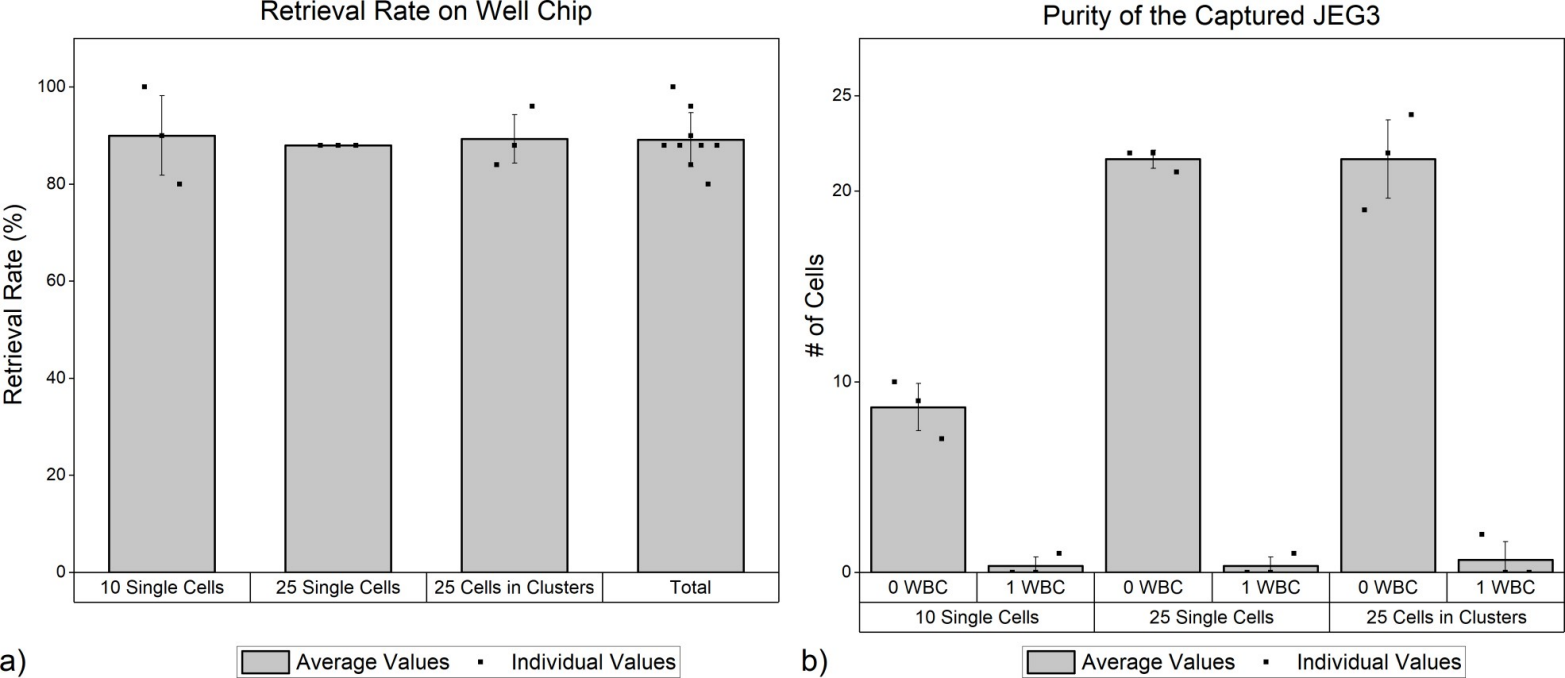
a) % Retrieval of the JEG3s. b) Purity of the captured JEG3s. # of cells refer to the target cells whereas 0 WBC and 1 WBC refer to the number of WBCs that are in the same well as the target cell.

In the experiments where we spiked 10 single cells into 8 ml of blood, our retrieval rates ranged from 80 to 100% with an average of 90% (see Fig 7A). In the experiments where we spiked 25 cells, our retrieval rates were consistently 88%. For the experiment where we spiked a combination of single cells and clusters, the successful retrieval rate varied from 84% to 96% with an average of 89.3%. (This set of experiments is denoted as “25 cells in clusters” in Fig 7A and shall be described with additional detail later). The overall retrieval yield between all of the aforementioned experiments was 89.1%. Lack of the CD45 stain on those cells that are positive for HLA-G/EpCAM/Hoechst, as well as the fact of consistently retrieving cells that are less than or equal to the number of spiked cells suggest that the experiments did not yield false positives.

We also studied the purity of the single cells we were able to retrieve (Fig 7B). The main contaminant in a typical rare cell detection experiment in blood is WBCs. Our system discards a great majority of the WBCs during the first (i.e. the porous chip) stage and then compartmentalizes the remaining WBCs into individual wells of the well chip to further enhance purity. Ideally, every well should be occupied by one cell only. Therefore, we investigated the extent of the imperfection in the system where a WBC accidentally co-occupies a well along with a target cell. These results are shown in Fig 7B for each set of experiments. We can define a ‘purity percentage’ as the number of target cells retrieved from a well, divided by the total number of cells in that same well. A 100% purity would indicate that a single target cell retrieved from a well had no WBCs with it. Between the 3 experiments with 10 single cells, where we were able to retrieve a total of 27 of the 30 spiked cells, 26 of these cells had 100% purity and 1 cell had 50% purity (it co-occupied a well with a WBC and was therefore retrieved along with it). Between the 3 experiments with 25 single cells we were able to retrieve a total of 66 of the 75 spiked cells; with 65 cells with 100% purity and 1 cell with 50% purity. Finally, for the experiments with 25 cells in clusters, we were able to retrieve 67 of the spiked 75 cells; with 65 cells with 100% purity and 2 cells, each with 50% purity. For all cases with 50% purity, the well had 1 target cell and 1 WBC in it. In total, 156/160 (98%) cells retrieved had 100% purity.

In addition to assessing the total number of cells retrieved and the purity of the cells retrieved, we also investigated the ability of the system in maintaining single cells as single cells and clusters as clusters. Additionally, we kept track of the dimeter of the wells the cells were retrieved from to confirm that we could indeed move larger clusters further downstream of the well chip, while keeping the single cells confined in the smaller wells upstream.

**Fig 8:**
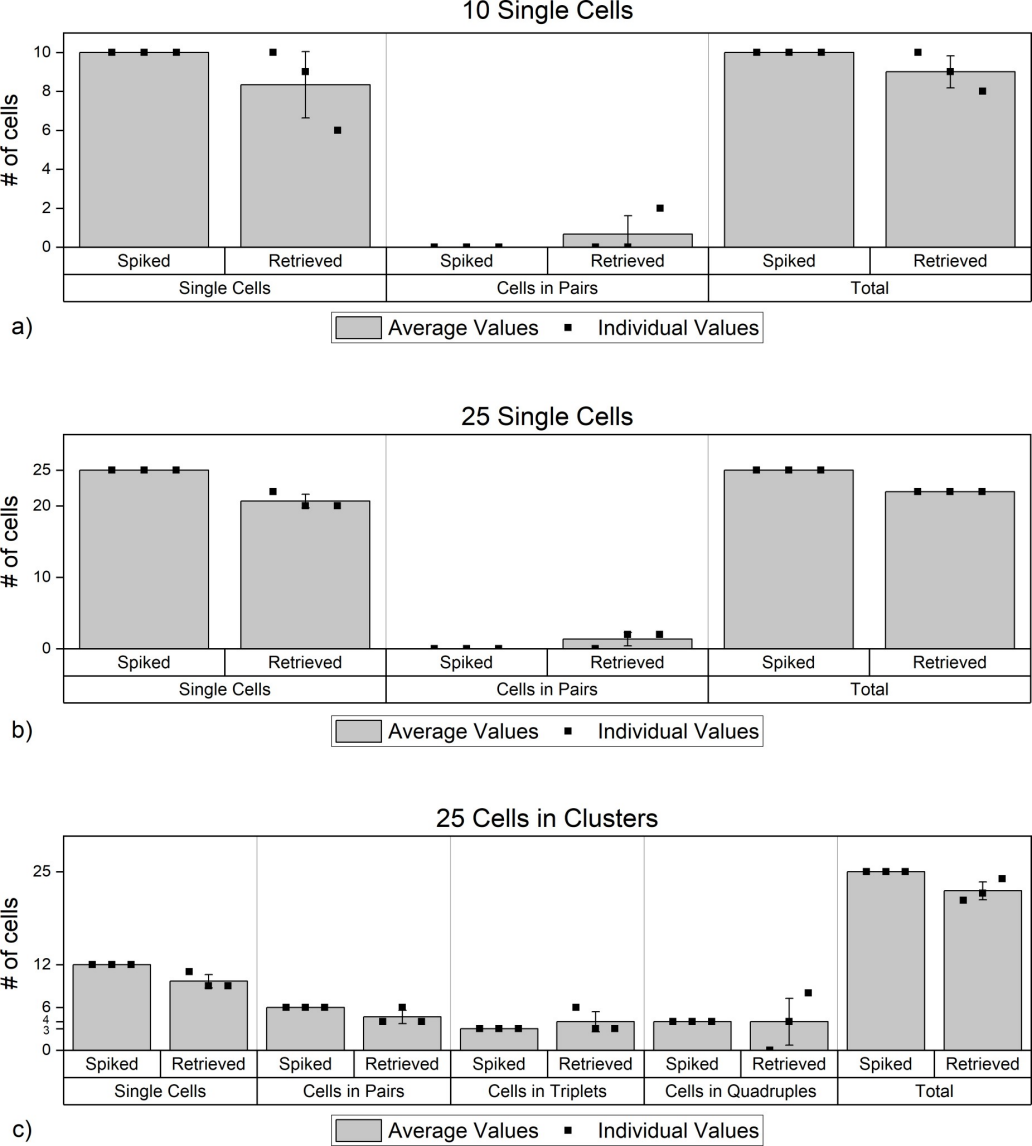
Cluster distribution data for experiments with a) 10 single cells, b) 25 single cells, c) 25 cells in clusters.

Between the 3 experiments with 10 spiked single cells, 25 out of the 27 cells that were retrieved were in single cells form and only 2 of these single cells fell into the same well and formed a “pair” (Fig 8A). For the experiments with 25 spiked single cells, 62/66 cells we retrieved were in single cells form and only 2 pairs were formed on the well chip (Fig 8B).

In each of the 3 experiments with “25 cells in clusters”, 12 single cells, 6 cells in pairs (i.e. 3 pairs), one triplet, and one quadruplet were spiked. Of the 12 single cells spiked, we were able to retrieve 9, 11, and 9 cells in each experiment. Of the 6 cells spiked in pairs (i.e. 3 pairs), we were able to retrieve 6, 4 and 4 cells (or 3, 2 and 2 pairs). The triplet that was spiked was retrieved as a triplet in 2 out of 3 experiments and an additional triplet was observed (i.e. 3, 6 and 3 cells in triplets were retrieved). The quadruple that was spiked was retrieved as is in one of the 3 experiments. In another experiment, no quadruple was observed in the end of the experiment. Yet in the 3^rd^ experiment, two quadruples were observed although only one quadruple was spiked (0,4, and 8 cells in quadruplets). No clusters larger than a quadruplet was observed (Fig 8C).

Even though a few clusters appeared “lost”, and a few “created”, the high percentage of the total number of cells retrieved (regardless of their cluster status) suggest that those clusters may have merely disintegrated into smaller clusters during the process and then combined with smaller clusters or single cells to co-occupy a well. For example, it is highly probable that a quadruple that was carefully aspirated from the culture medium as a quadruple could have disintegrated into smaller clusters during the incubation step after it was spiked into blood sample (which is rotated for 35 minutes) before it is sent into the device.

The formation of the extra clusters could also have happened at different stages of the experiments: while the cells were being incubated inside the blood tube, while they were pulled down to the porous chip, while they were being transferred from the porous chip to well chip, or while they were being dragged on the surface of the well chip by the magnet underneath. Such imperfections could be further mitigated by increasing the dimensions of the microchips and by using wider magnets to ensure that the cells are spread out further when they are on the surfaces of the microchips.

**Fig 9:**
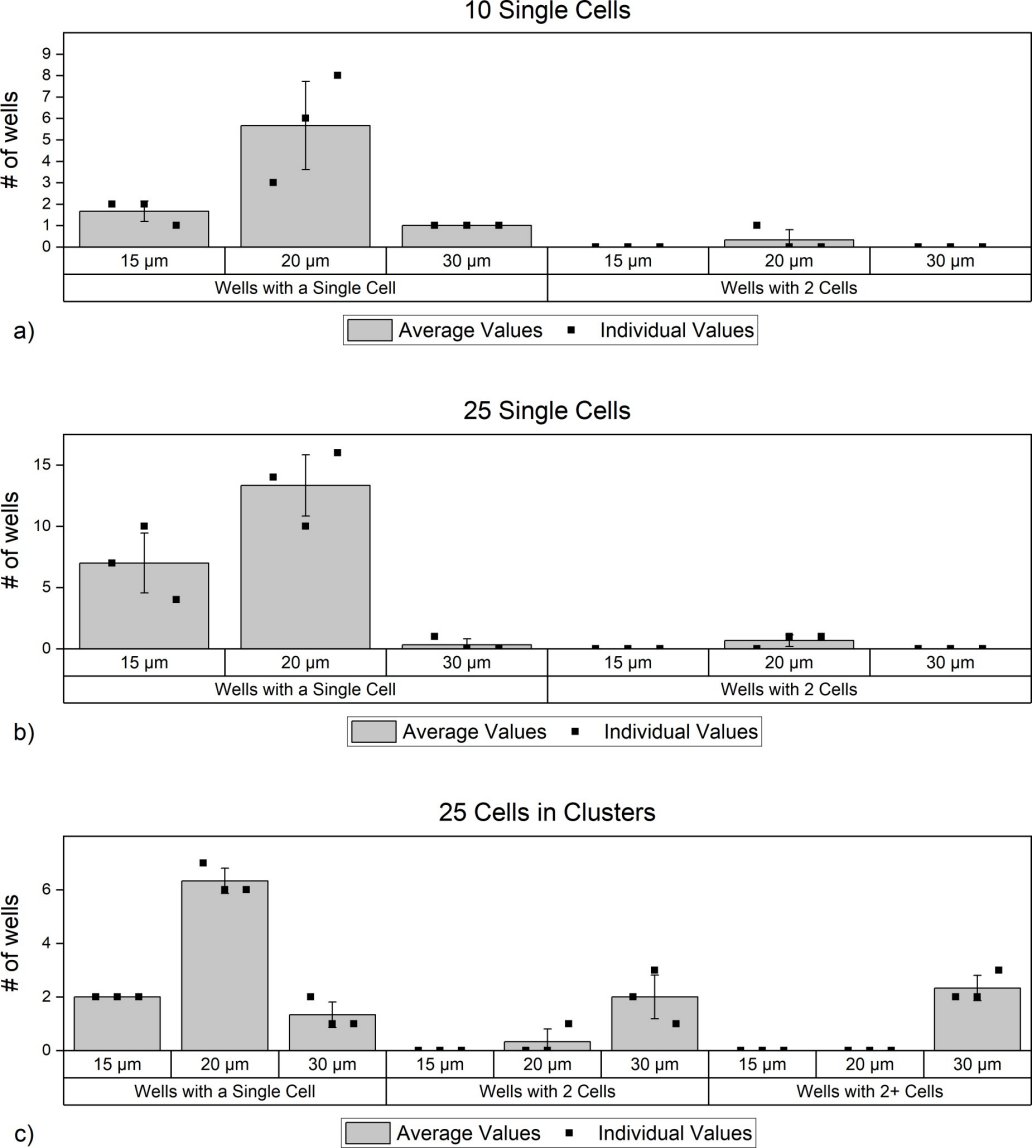
JEG3 distribution relative to well diameter for experiments with a) 10 single cells, b) 25 single cells, c) 25 cells in clusters.

Finally, we tracked the locations of the cells on the well chip. For the experiments with 10 single cells, the majority of the single cells were found in the 20 μm wells. Fewer cells landed in 15 μm wells (upstream of the 20 μm well) and only 1 cell in each experiment landed in a 30 μm well (Fig 9A). Only in one of the 3 experiments, was a pair encountered in a 20 μm well. It is possible that in this one case, a cell was fully inside the well, with its co-inhabitant partially sticking outside the well. In our experiments, we observed that as long as one of the cells fits inside a well, the pair will not move on the well chip with the movement of a magnet underneath.

For the case where 25 single cells were spiked as single cells, as well as the case where 25 cells were spiked in clusters, the results were similar to the 10 single cell experiments: more single cells in the 20 μm well, and fewer in 15 and 30 μm wells. (Fig 9B and 9C). As expected, in the experiment where 25 cells were spiked in clusters, cells in pairs and other clusters mostly settled in 30 μm wells (Fig 9C).

When we combine the data from 3 different experiments, we consistently observed that single cells mostly occupied 20 μm wells, while the pairs mostly occupied 20 μm and 30 μm wells. Clusters larger than pairs were all observed in wells with 30 μm diameter which demonstrates that those larger clusters were able to slide downstream on the well chip under the influence of the horizontally moving magnetic field.

## Conclusion

We have demonstrated the engineering and the proof of concept of a system consisting of 2 different micro chips used in sequence to enhance the purity in isolation of rare cells from blood. The first of these chips performs a bulk isolation where the majority of the unwanted cells are discarded. The second microchip, called the “well chip” then takes the cells captured by the first chip and further increases the purity by compartmentalizing them into wells where they can be retrieved in a controlled manner without interference by cells captured in other wells. The compartmentalization occurs by manipulating the cells on the well chip via a magnetic field that moves horizontally downstream and drags the cells until they encounter a well and fall in. Thanks to the robustness of the operation of the system, cells are compartmentalized simultaneously by manually moving a magnet downstream and parallel to the chip surface, eliminating the need to observe the movement of each cell sequentially. This unique design also inherently allows rapid separation of single cells from cell clusters by arranging well diameters to increase from up to downstream. We performed extensive characterization of the system to assess the overall capture yield, purity of the retrieved cells, as well as their distribution on the well chip. We were able to capture near 90% of the spiked cells, with 98% of the captured cells having 100% purity. We also demonstrated that an assay could be completed under 2.5 hours (from spiking into blood till the retrieval of a cell).

Our current efforts are focused on testing the system on capturing fetal trophoblasts from blood samples of pregnant women. We expect this system to be highly useful in a variety of research and clinical settings.

## Supporting information

S1 Video Separation of single cells from clusters. Using a magnet underneath the microchip (which applies a downward as well as a horizontal force on the cells), single cells are directed into smaller wells; whereas, larger clusters do not fit into the smaller wells and are moved towards an area with larger wells. Here, the magnet was deliberately moved slowly to clearly demonstrate the movement of the cells and clusters in real time (normally, this operation is much faster and need not be observed under a microscope).
(MP4)

S2 Video Retrieval of cells with a pipette. Targeted single cells as well as cell clusters are retrieved from wells without interference from any neighboring cells.
(MP4)
